# HIF‐Regulated Pannexin‐1 Channel Drives Luminal ATP Accumulation in Kidney Cysts

**DOI:** 10.1096/fj.202502847RR

**Published:** 2026-05-12

**Authors:** Kathrin Skoczynski, Julia Katharina Scholz, Raluca Ursu, Johannes Schödel, Steffen Grampp, Stephanie Naas, Victoria Lauer, Mario Schiffer, Maike Büttner‐Herold, Bjoern Buchholz, Andre Kraus

**Affiliations:** ^1^ Department of Physiology I, Institute of Physiology University of Regensburg Regensburg Germany; ^2^ Department of Internal Medicine 5—Hematology and Oncology Friedrich‐Alexander‐Universität Erlangen‐Nürnberg (FAU) and University Hospital Erlangen Germany; ^3^ Department of Nephrology and Hypertension Friedrich‐Alexander‐Universität Erlangen‐Nürnberg (FAU) and University Hospital Erlangen Germany; ^4^ Department of Nephropathology, Institute of Pathology Friedrich‐Alexander‐Universität Erlangen‐Nürnberg (FAU) and University Hospital Erlangen Germany

**Keywords:** ATP, cyst growth, HIF‐1α, Pannexin‐1, polycystic kidney disease

## Abstract

Autosomal dominant polycystic kidney disease (ADPKD) is marked by the progressive development of bilateral kidney cysts, leading to compression of intact surrounding tissue and a subsequent decline in kidney function. Accumulation of ATP within the cyst fluid significantly drives cyst expansion by activating purinergic receptors, thereby promoting calcium‐dependent chloride secretion. Moreover, the induction of hypoxia‐inducible factor 1α (HIF‐1α) in cyst‐lining epithelial cells further enhances chloride conductance. However, the mechanisms underlying ATP release into the cyst fluid and the role of HIF‐1α in cyst enlargement remain incompletely understood. Here, we demonstrate that the ATP‐release channel Pannexin‐1 is regulated by HIF‐1α in kidney tubular cells, leading to its prominent localization at the apical membrane of kidney cysts in both human and murine ADPKD kidney tissue. Apical ATP release is elevated in PKD1‐deficient cyst‐forming cells following pharmacological induction of HIF‐1α and attenuated by two Pannexin‐1 inhibitors, Probenecid and Brilliant Blue FCF (BB‐FCF). HIF‐dependent cyst growth in vitro is inhibited by basolateral application of the cell‐permeable Probenecid, whereas the cell‐impermeable BB‐FCF requires luminal application to exert its effect. In conclusion, HIF‐1α promotes Pannexin‐1 expression in cyst‐lining cells, facilitating directional ATP release into the cyst lumen and driving ATP‐dependent cyst expansion.

## Graphical abstract

Autosomal dominant polycystic kidney disease causes progressive cyst enlargement in the kidneys, leading to hypoxia and induction of HIF‐1α. Our study identifies HIF‐1α‐dependent transcriptional upregulation of Pannexin1 (PANX1) in the apical membrane of cyst epithelial cells, promoting ATP release into the cyst lumen. Established pathways show that luminal ATP stimulates P2Y2 receptors, leading to Ca^2+^‐dependent activation of the chloride channel TMEM16A, which drives cyst expansion. Brilliant Blue FCF and probenecid inhibit PANX1‐mediated ATP release and reduce cyst growth. Image was created in BioRender. Buchholz (2026) https://BioRender.com/ub9z5y9.

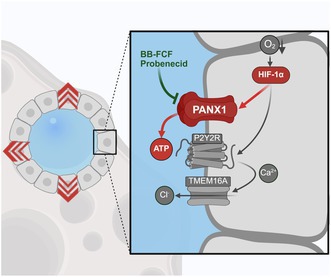

## 1 Introduction

Autosomal dominant polycystic kidney disease (ADPKD) is the most prevalent monogenic kidney disorder affecting approximately 1 in 1000 individuals and accounting for 5%–10% of end‐stage kidney disease cases [1]. The majority of patients harbor mutations in either the *PKD1* gene (encoding polycystin‐1, ~78%) or *PKD2* (encoding polycystin‐2, ~15%) [2]. ADPKD is characterized by the progressive development of multiple bilateral kidney cysts, which expand over time, leading to compression of adjacent intact parenchyma and a decline in kidney function [3].

Cyst enlargement is largely driven by chloride secretion into the cyst lumen. In addition to cAMP‐mediated chloride conductance, ATP‐dependent, calcium‐activated chloride secretion via TMEM16A (Anoctamin‐1) plays a substantial role in promoting cyst growth across in vitro, ex vivo, and in vivo models [4, 5]. ATP has been shown to accumulate in the cyst fluid at concentrations of up to 10 μM, which is sufficient to stimulate the purinergic receptor P2Y2R, which leads to activation of TMEM16A [6, 7]. Progressive cyst expansion induces local hypoxia, triggering the expression of the transcription factor hypoxia‐inducible factor 1α (HIF‐1α) in the cyst‐lining epithelium [8]. HIF‐1α has been shown to further exacerbate cyst growth by enhancing calcium‐activated chloride secretion in various ADPKD models, including a *Pkd1*‐deficient mouse model [9, 10].

Despite these findings, the mechanisms underlying ATP release into the cyst lumen, and the exact role of HIF‐1α in promoting chloride secretion, remain incompletely understood. In this study, we identify the ATP release channel Pannexin‐1 (PANX1) as a transcriptional target of HIF‐1α in kidney tubular cells. PANX1 is robustly expressed and apically localized in cyst‐lining cells of human and mouse ADPKD kidneys, facilitating directional ATP transport into the cyst lumen. Pharmacological induction of HIF‐1α increases PANX1‐mediated ATP release, which is effectively inhibited by the PANX1 blockers Probenecid and Brilliant Blue FCF (BB‐FCF). Notably, the cell‐impermeable BB‐FCF suppresses ATP release and cyst growth only when applied to the luminal side, further supporting a model of PANX1‐dependent apical ATP transport into the cyst fluid.

## 2 Methods

### 2.1 Human ADPKD Kidney Tissue

Nephrectomies of 29 patients (19 men and 10 women; further characteristics are summarized in Table 1) were obtained. Tissue was fixed immediately after nephrectomy in formaldehyde. Thereafter, a tissue microarray (TMA) was generated with a diameter of 2 mm per sample (*n* = 3 per patient) arranged over two object slides. All samples of the TMA were stained identically, and TMAs were scanned completely at 200× magnification by the use of a DM6000B fluorescence microscope (Leica, Wetzlar, Germany) and a Leica DFC 450C camera. Data in the corresponding graphs reflect the mean value of 3 samples of each patient. Collection and analysis of tissue samples were approved by the local ethics committee of the University Erlangen‐Nuernberg (reference number 22‐150‐D).

**TABLE 1:** Demographic and clinical characteristics at the time of nephrectomy.

Age	55.6 years (39.9–69.1)
Sex	Male: *n* = 19 Female: *n* = 10
Kidney replacement therapy (KRT)	Yes: *n* = 26 No: *n* = 3
Modality of KRT	Hemodialysis: *n* = 22 Kidney transplantation: *n* = 4 Peritoneal dialysis: *n* = 0 No RRT: *n* = 3
Duration of KRT	4.4 years (0.06–11.5)
eGFR (CKD‐EPI) of patients without KRT	12.7 mL/min/1.73 m^2^ (5.0–19.0)
Kidney volume of explanted kidney	2291 mL (628–9236)

### 2.2 Animals and Treatment

Animal experiments were approved by the local institutional review board and all animal experiments complied with the United Kingdom Animals Act, 1986, and associated guidelines, EU Directive 2010/63/EU for animal experiments. Experiments were approved by the local Ethics Committee of the Government of Unterfranken/Wuerzburg (Az. 55.2‐2532.1‐61/14). In this study, kidney sections of a tamoxifen‐inducible kidney epithelium‐specific *Pkd1* deletion mouse model crossed with a *Hif‐1α* knock‐out murine model were used. Generation of the tamoxifen‐inducible, kidney epithelium‐specific *Pkd1*‐deletion mouse model carrying the loxP‐flanked conditional alleles of *Pkd1* (Ksp*CreER* ^T2^;*Pkd1* ^lox;lox^) has been described previously [11]. Upon administration of tamoxifen to the mice, a genomic fragment containing exons 2–11 of the Pkd1 gene is specifically deleted in kidney epithelial cells inducing cyst formation. Ksp*CreER* ^T2^;*Pkd1* ^lox;lox^ mice were crossed with mice carrying loxP‐flanked alleles of *Hif‐1α* (*Hif‐1α* ^lox;lox^) as described previously in order to receive tamoxifen‐inducible, kidney epithelium‐specific deletion of *Pkd1* and *Hif‐1α* (Ksp*CreER* ^T2^;*Pkd1* ^lox;lox^;*Hif‐1α* ^lox;lox^) [10]. Genotyping was carried out by PCR on tail DNA samples as described previously [10]. Gene deletion was induced by daily intraperitoneal administration of tamoxifen (2 mg/kg body weight) dissolved in 5% ethanol and 95% neutral oil from postnatal days (PN) 35–37 to induce a slow disease progression in male mice. Ksp*CreER* ^T2^;*Pkd1* ^lox;lox^ mice that received vehicle only (without tamoxifen) at the same time points served as controls. PN 35–37 induced Ksp*CreER* ^T2^;*Pkd1* ^lox;lox^ mice (males, *n* = 7) and Ksp*CreER* ^T2^;*Pkd1* ^lox;lox^;*Hif‐1α* ^lox; lox^ mice (male: *n* = 5) received the HIF‐stabilizer 2‐(1‐chloro‐4‐hydroxyisoquinoline‐3‐carboxamido) acetate (ICA) by intraperitoneal injection (40 mg/kg body weight; dissolved in 5% DMSO and 95% 0.5 M Tris) 5 days per week for 12 weeks and were compared with Ksp*CreER* ^T2^;*Pkd1* ^lox;lox^ mice (males, *n* = 7) and Ksp*CreER* ^T2^;*Pkd1* ^lox;lox^;*Hif‐1α* ^lox;lox^ mice (males, *n* = 6) that received vehicle (with no ICA). Noninduced but vehicle‐treated Ksp*CreER* ^T2^;*Pkd1* ^lox;lox^ mice (males, *n* = 5) served as controls. PN 35–37‐induced animals were observed for 12 weeks. Thereafter, animals were sacrificed, and kidneys were harvested. The mice of which samples were utilized for this study were characterized for their cystic index as well as serum creatinine levels in our previous paper [10].

### 2.3 Immunohistochemistry and Antibodies

Transverse kidney sections (2 μm thickness) were subjected to immunohistochemical staining to detect PANX1 expression. A polyclonal rabbit anti‐PANX1 antibody (Abcam, ab139715; 1:500 dilution), followed by a biotinylated secondary goat anti‐rabbit IgG antibody (VEC‐BA‐1000; Vector Laboratories; 1:500 dilution), was used to label the target protein. Signal amplification was achieved sequentially using a catalyzed system (VECTASTAIN Elite ABC‐HRP Kit; Vector Laboratories, USA), followed by tyramide‐based biotin amplification (TSA Plus Biotin Kit; Akoya Biosciences, USA) and subsequent Streptavidin‐HRP conjugation (Abcam, ab64296). Since the polyclonal antibody produced unspecific nuclear staining in human ADPKD tissue (Figures S4–S6), human samples were additionally stained using a recombinant rabbit monoclonal anti‐PANX1 antibody (Thermo Fisher Scientific, MA5‐50140; 1:200 dilution). After deparaffinization and rehydration, sections were incubated overnight at 4°C with the monoclonal antibody, followed by a 30‐min incubation with a biotinylated goat anti‐rabbit IgG secondary antibody (Vector Laboratories, BA‐1000; 1:500 dilution). Signal amplification was performed using the VECTASTAIN ABC‐HRP Peroxidase Kit for 30 min, followed by Streptavidin‐HRP (Abcam, ab64296) for an additional 30 min. Signal visualization in all protocols was conducted using the chromogenic substrate 3,3′‐Diaminobenzidine (DAB). Finally, stained sections were examined using a DM6000B microscope (Leica Microsystems), and images were captured with a Leica DFC450C camera.

### 2.4 Quantification of Immunohistochemical Signals

Six random photos were taken from the cortex of each mouse kidney at a 200× magnification. TMA samples were analyzed entirely. Immunohistochemical signals of PANX1 were analyzed as described previously [10]. Briefly, a color deconvolution algorithm (ImageJ V.1.48) was applied to dissect the different signals followed by binarization and particle analysis to obtain the ratio of positive area per total tissue area. Thereafter, mean values from the six photos were calculated for each kidney. Values obtained from TMA samples reflected the entire tissue and the mean of three samples from one patient was calculated.

### 2.5 Human Primary Kidney Tubule Cells

Human primary tubular epithelial cells (hPTC) from male and female patients were isolated from kidney cortical tissues collected from healthy parts of tumor nephrectomies. Isolated cells represent a mixture of proximal and distal tubular cells with a ratio of approximately 50:50%, as described previously [12]. Isolation of human cells was approved by the local ethics committee (Reference numbers 329_16B and 542_20Bc, Ethik‐Kommission der Medizinischen Fakultät der Friedrich‐Alexander Universität Erlangen‐Nürnberg). Cortex tissue was cut into 1 mm^3^ pieces and digested with collagenase type II (Gibco, Karlsruhe, Germany) and DNase I grade II (Roche Diagnostics, Mannheim, Germany) for 60 min. Then, the cell suspension was sieved through 100 and 70 μm meshes, and cells were seeded in epithelial cell selective medium (DMEM/Ham's F12 medium containing 2 mM L‐glutamine, 100 U/mL penicillin, 100 mg/mL streptomycin, insulin‐transferrin‐selenium supplement, 10 ng/mL epidermal growth factor, 36 ng/mL hydrocortisone and 4 pg/mL triiodothyronine) in the presence of 0.5% fetal calf serum (FCS). After 1–2 days, the medium was replaced by FCS‐free medium. For HIF stabilization, cells were incubated for 16 h with dimethyloxalylglycine (DMOG; Sigma Aldrich) in a final concentration of 1 mM. The DMOG concentration (1 mM) was chosen based on previously published dose–response experiments in primary human tubular epithelial cells [13], which demonstrated robust HIF‐1α stabilization and HIF target gene induction at this concentration without evidence of cytotoxicity within the relevant incubation period. HIF‐1α protein levels peak at approximately 16 h under these conditions, which was therefore used as the standard incubation time in this study.

### 2.6 Chromatin Immunoprecipitations

HIF‐1α and HIF‐1β ChIP qPCR and ChIP‐seq data from human primary tubular epithelial cells were generated in previous work [14]. Data are available at GEO (GSE101064). BigWig files were visualized using the IGV viewer (IGV_2.19.4).

### 2.7 Real‐Time PCR

DNA was prepared using peqGOLD TriFast (peqlab/VWR International GmbH, Erlangen, Germany) according to the manufacturer's instructions. SYBR Green‐based real‐time PCR was performed using StepOnePlus (Applied Biosystems, Foster City, CA). mRNA expression levels were normalized to 18S rRNA using the ΔΔ*C*_t_ method. Primer sequences are listed in Table S1.

### 2.8 siRNA of HIF‐1α and HIF‐1β

The use of siRNA directed against HIF‐1α and HIF‐1β or a non‐targeting siRNA has been described previously [15]. siRNAs were transfected using Lipofectamin3000 reagent (Thermo Fisher Scientific Inc., Erlangen, Germany) with a final concentration of 40 nM. siRNA sequences are listed in Table S2.

### 2.9 PKD1‐Deficient plMDCK Cells

Generation of *PKD1*‐deficient plMDCK cells has been described earlier [5, 16]. In short, a subclone of MDCK cells was used that resembles principal cells of the collecting duct (C7 clone, kind gift from Prof. Hans Oberleithner). For genome editing, pSpCas9(BB)‐2A‐puro‐vectors (PX459) V2.0 (Addgene, Watertown, MA, USA) were used to generate a *PKD1* ^−/−^ cell line. The guide RNA was designed according to the algorithms provided by the Zhang laboratory (http://crispr.mit.edu/), which provided a quality score of 92. After ligation of the DNA‐oligonucleotides with the vectors, 10^6^ cells were transfected using 10 μL polyethylenimine and 4 μg of the vector. After 24 h, cells were incubated with puromycine (3.5 μg/mL) for 48 h. Primers used for generation of the *PKD1* ^−/−^ knockout cell line have been published before [16]. Clones of cells were generated by dilution. For mutation screens, genomic DNA of single‐cell clones was isolated and the CRISPR/Cas9 target region was amplified by PCR. Products were resolved by polyacrylamide gel electrophoresis. Genomic DNA of potential cell clones was amplified by PCR and cloned into pGL3 vector (Promega, Madison, WI, USA) and subjected to Sanger sequencing (Eurofins, Nuernberg, Germany). The two main potential off‐targets in DNA regions were tested for off‐target effects by PCR amplification and polyacrylamide gel electrophoresis analysis. No off‐target effects were detected.

### 2.10 Western Blot

Principal‐like MDCK cells either competent for *PKD1* (wildtype and clone *PKD1* ^+/+^ #1) or deficient for *PKD1* (*PKD1* ^−/−^ #1 and #2) were previously characterized [5] and cultured under standard conditions (37°C, 21% O_2_, 5% CO_2_) in modified minimum essential medium (MEM) containing Earl's balanced salt solution. The medium was supplemented with 2 mM L‐glutamine, 10% heat‐inactivated fetal calf serum (FCS), 50 U/mL penicillin, and 50 μg/mL streptomycin. ICA was used at 10 μM, a concentration previously shown to induce robust HIF‐1α stabilization in plMDCK cyst models [9] and which does not differ in efficacy from 100 μM in plMDCK cells (see Figure 4B). This concentration is therefore consistent with our established protocols for longer incubation periods. In addition, 5 μM acriflavine, a widely used small molecule HIF inhibitor, was applied in accordance with studies demonstrating effective HIF 1 inhibition at low micromolar doses in epithelial cell models without inducing cytotoxicity [17].

Total cellular proteins were extracted using lysis buffer containing 6.65 M urea, 10% glycerol, 10 mM Tris–HCl (pH 6.8), 1% SDS, 5 mM dithiothreitol (DTT), and a complete protease inhibitor cocktail (Roche), followed by sonication. Total cellular protein content was determined according to the method of Neuhoff et al. [18]. One microliter of each protein sample was spotted onto a cellulose acetate filter (Sarstedt, Nümbrecht, Germany) and incubated for 2 min in Neuhoff staining solution (0.5% Amido Black 10B [w/v], 90% methanol [v/v], 10% acetic acid [v/v]). Filters were subsequently destained in Neuhoff destaining solution (90% methanol [v/v], 10% acetic acid [v/v]) and air dried. The dried filters were transferred to test tubes containing 1 mL DMSO and dissolved by shaking for 45 min. Aliquots of 100 μL were then transferred to a 96 well plate, and absorbance was measured using a microplate photometer (Promega GmbH, Walldorf, Germany). For PANX1 immunodetection, 50 μg of protein per sample were separated and probed using a polyclonal rabbit anti‐PANX1 primary antibody (Abcam, ab139715; 1:1000 dilution) or a primary anti‐HIF‐1α antibody (Cayman Chemicals, Ann Arbor, MI; 1:1000). Detection was performed using an HRP‐conjugated polyclonal goat anti‐rabbit secondary antibody (Dako P0399; 1:10 000 dilution). β‐actin or Vinculin were used as a loading control and detected with an HRP‐coupled rabbit antibody (Sigma‐Aldrich; 1:30 000 dilution).

### 2.11 ATP Measurements

*PKD1* ^−/−^ #1 cells were seeded on Millicell hanging cell culture inserts (catalog no. PIHP01250; Merck, Darmstadt, Germany) at a density of 100 000 cells per insert using phenol red‐free medium. After 72 h of cultivation, transepithelial electrical resistance (TEER) was measured using the EVOM2 resistance meter (World Precision Instruments, Friedberg, Germany) to assess monolayer integrity. For inserts exhibiting high confluency and stable resistance values, the apical and basal media were subsequently replaced with fresh medium containing the respective experimental supplements. BB‐FCF was applied at a concentration of 100 μM, a dose widely used in the literature to ensure selective extracellular inhibition of PANX1 without affecting P2X7 or other purinergic receptors [19]. After an additional 24 h of incubation, both apical and basal media were collected, and ATP concentrations were determined using the BacTiter‐Glo Microbial Cell Viability Assay (catalog no. G8230; Promega, Madison, WI, USA) according to the manufacturer's instructions.

### 2.12 In Vitro Cyst Model

In vitro cyst assays using principal‐like MDCK cells deficient for *PKD1* (*PKD1* ^−/−^ #1) were performed as described previously [5]. In brief, cells were resuspended as a single‐cell suspension in type I collagen and transferred to 24‐well plates (3 wells per condition). Probenecid or BB‐FCF was added to the medium at concentrations of 250, 500, 750, and 1000 μM on day 0 and medium was exchanged every 48 h. All substances were purchased from Sigma (Taufkirchen, Germany). After 5 days, images were taken and analyzed in an automated fashion by capturing diameters of all spherical cysts and calculation of the volumes according to 4/3*πr* ^3^ as described previously [16].

Cysts were microinjected with either the PANX1 inhibitor Brilliant Blue FCF (BB‐FCF) or Brilliant Cresyl Blue (BCB) as control and subsequently monitored via live imaging. BB‐FCF was applied at a concentration of 100 μM, dissolved in phosphate‐buffered saline (PBS) containing 8.7 μM BCB for visualization. The mean volume of injected cysts was 21.8 nL (range: 8.0–48.7 nL). A fixed injection volume of 1 nL resulted in final luminal concentrations of 4.6 μM BB‐FCF (range: 2.1–12.5 μM). Notably, the reported IC_50_ of BB‐FCF for PANX1 is 0.27 μM [19]. For control conditions, cysts were microinjected with 1 nL PBS containing 8.7 μM BCB. Since cysts consistently exhibited a brief post‐injection stabilization phase dominated by mechanical effects, the time point 10 h post‐injection was defined as the earliest reproducible and biologically meaningful reference time point (T1). In each experiment, cyst diameter and corresponding volume were quantified between T1 and a subsequent time point at 72 h (T2) in a blinded manner.

### 2.13 Statistical Analysis

Bars show mean ± SEM. Each point represents one individual animal. Differences among groups were analyzed using one‐way ANOVA, followed by Tukey's test for multiple comparisons. An unpaired *t*‐test was applied to compare the differences between two groups. Wilcoxon signed‐rank test for column statistics was used for relative values. *p* < 0.05 was considered statistically significant.

## 3 Results

### 3.1 HIF‐1α Binds to Intragenic Elements of PANX1 and Modulates Its Expression in Human Primary Kidney Tubular Cells

**FIGURE 1:**
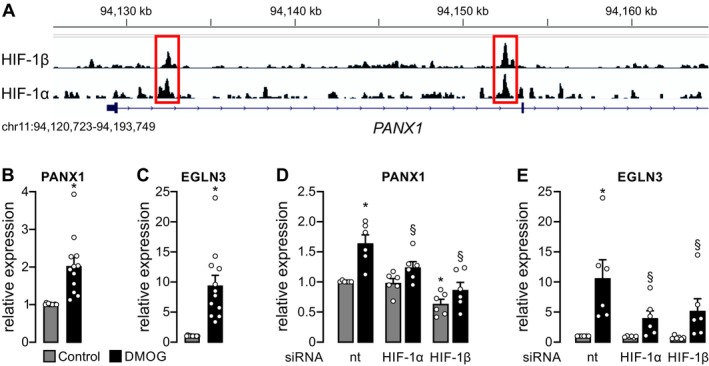
HIF interacts with intragenic regions of *PANX1* and regulates its expression in human primary tubular cells. (A) HIF‐1α and HIF‐1ß ChIP‐seq tracks from human primary epithelial tubular cells (hPTC) exposed to 1 mM of the HIF‐α prolyl‐4‐hydroxylase domain (PHD) enzyme inhibitor dimethyloxalylglycine (DMOG) for 16 h. Shown are the genomic loci of *PANX1*. Conserved binding of both HIF subunits is highlighted in red and occurs within intragenic regions of *PANX1*. (B) hPTC from *n* = 12 individuals were cultured under control condition or incubated with 1 mM DMOG for 16 h. PANX1 mRNA expression was measured by qPCR. (C) mRNA expression of EGLN3 (a well‐characterized target gene of HIF) of the cells described in (B) was analyzed in comparison with control cells. *Significant compared with control. (D) hPTC from *n* = 6 individuals were cultured under control condition or incubated with 1 mM DMOG for 16 h in the presence and absence of siRNA directed against HIF‐1α, HIF‐1β or a non‐targeting (nt) siRNA serving as control. 32 h after transfection, cells were exposed to control conditions or 1 mM DMOG for 16 h. PANX1 mRNA expression is depicted in comparison with control cells under untreated control condition (set = 100%). (E) mRNA expression of EGLN3 of the cells described in (D) was analyzed in comparison with control cells in the presence of non‐targeting (nt) siRNA (set = 100%). *Significant compared with nt control condition; ^§^significant compared with nt +DMOG.

Hypoxia has been shown to promote ATP release in various non‐epithelial cells mainly by vesicular nucleotide release [20]. In kidney tubule cells, Connexin 30 (Cx30), Pannexin‐1, and LRRC8A have been suggested as candidates to mediate apical ATP release [21]. We therefore screened available HIF ChIP‐seq data from human primary kidney tubule cells (hPTC) exposed to the HIF‐stabilizer dimethyloxalylglycine (DMOG) for HIF‐1α and HIF‐1β binding events in intragenic regions of the corresponding genes. For *PANX1*, but not the other candidates, we detected two HIF‐binding events in intronic regions of the gene suggesting potential regulation of *PANX1* by HIF‐1 (Figure 1A; Figure S1A,B). HIF‐binding to the well‐characterized transcriptional intronic enhancer of EGLN3 served as a positive control (Figure S1C). These sites also meet our predefined criteria for HIF target loci, as we only consider regions with overlapping HIF‐1α and HIF‐1β (ARNT) binding peaks. Of note, TMEM16A (ANO1) does not show HIF‐1α binding (Figure S1D), indicating that the HIF‐dependent increase in calcium‐activated chloride secretion is unlikely to result from direct transcriptional regulation of TMEM16A but rather from enhanced upstream stimulation or activation.

To explore regulation of *PANX1* expression by HIF‐1, we used freshly isolated hPTC and exposed them to DMOG for 16 h to pharmacologically stabilize HIF‐1α. We measured a moderate but significant increase of *PANX1* mRNA levels to 2.03 ± 0.77‐fold by qPCR upon application of DMOG when compared with standard culture conditions (Figure 1B). *EGLN3*, a well‐characterized direct target gene of HIF, served as positive control (Figure 1C). We next used siRNA directed against human HIF‐1α, the HIF‐α isoform expressed in tubule cells, or its dimerization partner HIF‐1β to compare the impact of DMOG with control‐treated hPTC (Figure 1D). DMOG treatment led to a significant increase of *PANX1* mRNA expression in control‐treated cells, but this effect was significantly abrogated in cells transfected with siRNA directed against HIF‐1α or HIF‐1β (Figure 1D). Again, *EGLN3* served as positive control (Figure 1E). This indicates HIF‐1α‐dependent regulation of *PANX1* on a transcriptional level in hPTC.

### 3.2 PANX1 Is Expressed in Cysts of Human ADPKD Nephrectomies

**FIGURE 2:**
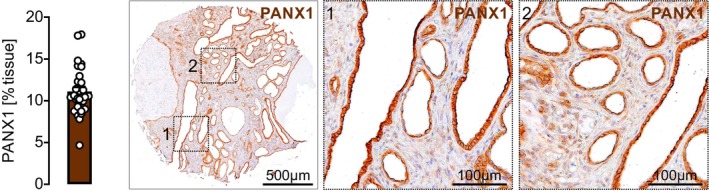
PANX1 is expressed in cysts of human ADPKD nephrectomies. Kidney specimens of 29 ADPKD patients (male: *N* = 19; female: *N* = 10) undergoing nephrectomy were obtained and analyzed. Further clinical characteristics are summarized in Table 1. Quantification of PANX1 staining expressed as positive staining per tissue area. Each data point represents the mean value of 3 samples per patient. Representative tissue microarray sample stained for PANX1 (brown). Squares mark corresponding magnifications on the right (1, 2), which reveal PANX1 staining predominantly at the luminal site of the cyst‐lining epithelium.

We next examined kidney tissue from 29 ADPKD patients who had undergone nephrectomy, assessing PANX1 protein expression and its subcellular localization. A higher proportion of male patients underwent nephrectomy. The majority were diagnosed with end‐stage kidney disease, receiving kidney replacement therapy, predominantly via hemodialysis. Demographic and clinical characteristics at the time of nephrectomy are detailed in Table 1. PANX1 expression was consistently detected across all specimens, with prominent localization to the cyst‐lining epithelial cells (Figure 2; Figures S2 and S3). To ensure robust detection of PANX1 in human tissue, we evaluated two independent antibodies. The polyclonal antibody yielded the expected epithelial staining pattern but also showed additional nuclear signal in human ADPKD samples (Figures S4–S6). Therefore, PANX1 localization was validated using a recombinant monoclonal antibody, which produced a clear apical membrane and cytoplasmic signal in cyst‐lining epithelial cells, consistent with the distribution shown in Figure 2.

### 3.3 HIF‐1α Regulates PANX1 Expression in the Cyst Epithelium of a Pkd1‐Deficient Mouse Model

To assess PANX1 expression in cyst‐lining epithelial cells and examine its regulation by HIF‐1α in vivo, we analyzed kidneys from an orthologous ADPKD mouse model. We employed a tamoxifen‐inducible, kidney epithelium‐specific *Pkd1* knockout model (Ksp*CreER* ^T2^;*Pkd1* ^lox;lox^) and crossed it with floxed *Hif‐1α* mice (*Hif‐1α* ^lox;lox^) to generate mice with conditional, tubule‐specific deletion of both *Pkd1* and *Hif‐1α*.

**FIGURE 3:**
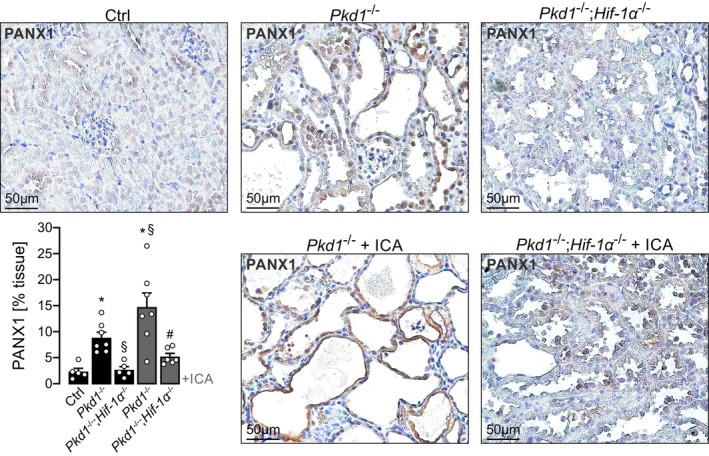
PANX1 is expressed in cysts of a *Pkd1* orthologous mouse model and regulated by HIF‐1α. PANX1 is expressed in a HIF‐1α‐dependent manner in cyst‐lining cells of an ADPKD mouse model. Tamoxifen was applied at postnatal days 35–37 to induce tubule‐specific deletion of *Pkd1* in Ksp*CreER* ^T2^;*Pkd1* ^lox;lox^ (*Pkd1* ^−/−^; *n* = 7) mice. In parallel, genetic deletion was induced in Ksp*CreER* ^T2^;*Pkd1* ^lox;lox^;*Hif‐1α* ^lox/lox^ (*Pkd1* ^−/−^;*Hif‐1α* ^−/−^; *n* = 5) mice to receive tubular codeletion of *Pkd1* and *Hif‐1α*. In addition, mice were either treated with the PHD inhibitor 2‐(1‐chloro‐4‐hydroxyisoquinoline‐3‐carboxamido) acetate (*Pkd1* ^−/−^ +ICA; *n* = 7); (*Pkd1* ^−/−^;*Hif‐1α* ^−/−^ +ICA; *n* = 6) or its vehicle for 12 weeks. Non‐induced mice served as controls (Ctrl; *n* = 5). Kidneys from Pkd1^−/−^ mice showed a significant increase of PANX1 expression compared to control where PANX1 was hardly detectable. Codeletion of *Hif‐1α* in addition to *Pkd1* (*Pkd1* ^−/−^;*Hif‐1α* ^−/−^) resulted in lower expression of PANX1 compared to *Pkd1* ^−/−^. Application of ICA significantly increased PANX1 expression in *Pkd1* ^−/−^ kidneys (*Pkd1* ^−/−^ +ICA), which was significantly abrogated in double knockout mice (*Pkd1* ^−/−^;*Hif‐1α* ^−/−^ +ICA). *Significant compared with Ctrl. ^§^Significant compared with *Pkd1* ^−/−^. ^#^Significant compared with *Pkd1* ^−/−^ +ICA.

Deletion of *Pkd1* was induced between postnatal days 35–37, resulting in a moderate polycystic phenotype 12 weeks post‐induction, accompanied by a marked upregulation of PANX1 expression compared to control kidneys (Figure 3). Simultaneous deletion of *Hif‐1α* in *Pkd1*‐deficient mice not only ameliorated cyst formation (as previously published [10]) but also significantly reduced PANX1 expression (Figure 3). Conversely, pharmacological stabilization of HIF‐1α using the prolyl‐hydroxylase inhibitor 2‐(1‐chloro‐4‐hydroxyisoquinoline‐3‐carboxamido) acetate (ICA) led to exacerbated cyst growth and pronounced PANX1 upregulation in *Pkd1*‐deficient kidneys. Notably, both ICA‐induced cyst enlargement and PANX1 upregulation were abolished by tubule‐specific deletion of *Hif‐1α*.

Collectively, these findings provide in vivo evidence that PANX1 is transcriptionally regulated by HIF‐1α and contributes to cyst expansion in *Pkd1*‐deficient kidneys.

### 3.4 Deletion of PKD1 Results in Increase of PANX1 Protein in Cyst‐Forming Tubule Cells

**FIGURE 4:**
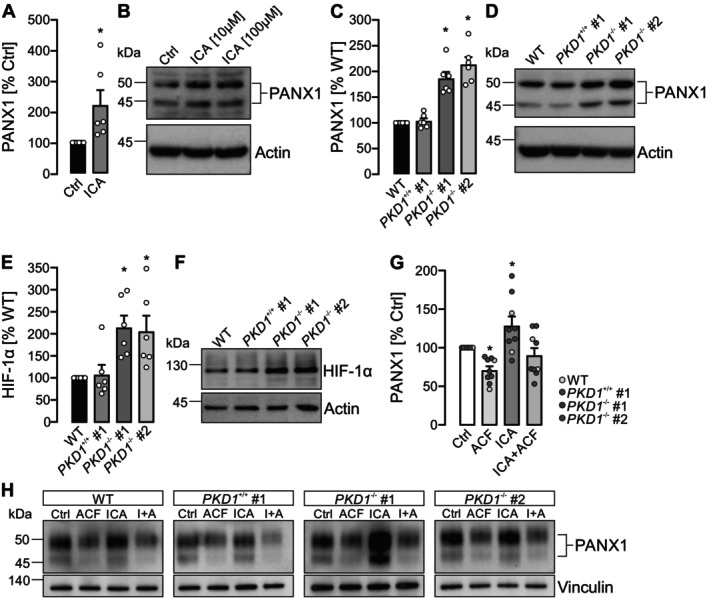
HIF and loss of *PKD1* increases PANX1 protein levels in cyst‐forming kidney tubule cells. (A) Principal‐like MDCK (plMDCK) cells were incubated under control conditions (Ctrl) or stimulated with 10 μM ICA. PANX1 protein expression was quantified in six independent experiments, normalized to β‐Actin, and expressed relative to Ctrl (set to 100%). (B) Representative Western blot corresponding to panel (A). The two bands indicate glycosylated (upper) and non‐glycosylated (lower) PANX1 isoforms. β‐Actin served as loading control. Increasing ICA concentration to 100 μM had no additional effect. (C) PANX1 protein levels were assessed in wild‐type plMDCK cells (WT), a *PKD1*‐competent clone (*PKD1* ^+/+^ #1), and two PKD1‐deficient clones (*PKD1* ^−/−^ #1 and #2). Data represent mean expression from *n* = 6 experiments, normalized to β‐Actin and set to 100% in WT. Loss of *PKD1* was associated with elevated PANX1 expression. (D) Representative blot corresponding to panel (C). (E) HIF‐1α protein levels were determined in the plMDCK cells described in (C). Data represent mean expression from *n* = 6 experiments, normalized to β‐Actin and set to 100% in WT. *PKD1*‐deficient clones showed increased HIF‐1α expression. (F) Representative blot corresponding to panel (E). (G) plMDCK cells described in (C) were incubated under control conditions (Ctrl) or stimulated with 5 μM of the HIF‐inhibitor acriflavine (ACF), 10 μM ICA, or the combination of both (I + A). PANX1 protein expression was quantified in nine independent experiments (*n* = 5 *PKD1*‐competent, and *n* = 4 *PKD1*‐deficient samples), normalized to vinculin, and expressed relative to Ctrl (set to 100%). The data demonstrate HIF‐dependent regulation of PANX1. (H) Representative blots corresponding to panel (G). *Statistical significance relative to Ctrl (A, G) or *PKD1* ^+/+^ (C, E).

To further investigate the functional relevance of our findings, we employed a principal‐like (pl) MDCK cell subclone, which closely models the principal cells of the collecting duct—the predominant site of cyst formation in ADPKD [22]. Treatment with the prolyl‐hydroxylase inhibitor ICA resulted in upregulation of PANX1 expression (Figure 4A,B), consistent with observations from human tubular epithelial cells and in vivo mouse models. To assess the impact of *PKD1* deletion on PANX1 levels, we generated *PKD1*‐deficient plMDCK cell clones using CRISPR/Cas9 and compared them to wild‐type and *PKD1*‐competent controls. Loss of *PKD1* led to a significant increase in PANX1 protein expression (Figure 4C,D). We next examined HIF‐1α protein abundance in *PKD1*‐deficient cells. Both *PKD1* ^−/−^ clones displayed elevated HIF‐1α levels compared to *PKD1*‐competent controls (Figure 4E,F), suggesting that increased HIF signaling may contribute to the higher PANX1 expression in the absence of *PKD1*. To functionally test this relationship, we treated wild‐type, *PKD1*‐competent, and *PKD1*‐deficient cells with the HIF‐1 inhibitor acriflavine, with ICA, or with the combination of ICA and acriflavine. HIF‐1 inhibition reduced PANX1 expression across all genotypes, whereas ICA induced a marked increase in PANX1 levels (Figure 4G,H). Importantly, acriflavine prevented the ICA‐dependent upregulation of PANX1, supporting a HIF‐1‐dependent mechanism of PANX1 regulation in the plMDCK cells. Based on these findings, we selected the *PKD1* ^−/−^ #1 clone for subsequent analyses of PANX1‐mediated ATP release and cyst growth.

### 3.5 PKD1‐Deficient Tubule Cells Release ATP Apically Which Is Increased by HIF Stabilization and Inhibited by PANX1 Inhibitors

To assess apical ATP release from *PKD1*‐deficient tubular cells, we seeded *PKD1* ^−/−^ #1 clone cells onto permeable supports and evaluated monolayer integrity by measuring transepithelial resistance, which averaged 12 349 ± 789 Ω/cm^2^ across experiments. ATP concentrations were subsequently measured in both apical and basolateral media following various interventions.

**FIGURE 5:**
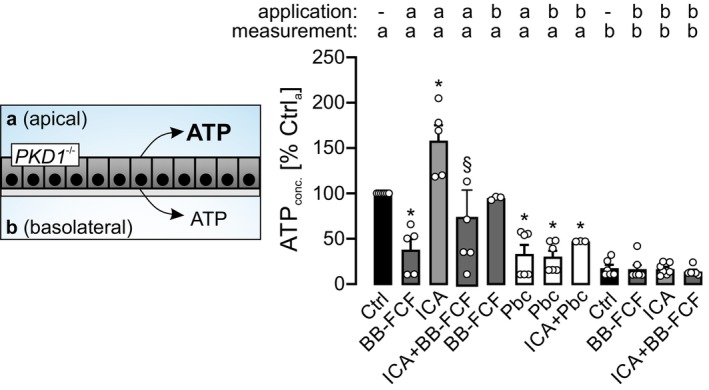
Apical ATP release in *PKD1*‐deficient tubule cells is enhanced by HIF stabilization and inhibited by PANX1 blockers. *PKD1* ^−/−^ #1 cells were cultured on permeable filters permitting compound application and ATP measurements at both the apical (a) and basolateral (b) compartments. Monolayer integrity was confirmed via transepithelial resistance. Apical ATP release under control conditions was quantified (*n* = 7) and set to 100% within each experiment. Apical application of BB‐FCF (100 μM; *n* = 5) significantly reduced ATP release compared to control. ICA stimulation (10 μM; *n* = 5) increased apical ATP levels, which was counteracted by apical co‐application of BB‐FCF (100 μM; *n* = 5). In contrast, basolateral BB‐FCF (100 μM; *n* = 3) did not affect apical ATP release. Probenecid (500 μM), whether applied apically (*n* = 5) or basolaterally (*n* = 5), significantly suppressed apical ATP release and abolished ICA‐induced ATP increase (*n* = 3). Basolateral ATP concentrations were lower than apical values under control conditions (*n* = 6) and remained unaffected by BB‐FCF (100 μM; *n* = 7), ICA (*n* = 7), or their combination (*n* = 6), when applied basolaterally. *Significant compared with apical ATP concentration under control conditions (Ctrl ‐/a). ^§^Significant compared with apical ATP concentration in the presence of ICA (ICA a/a).

Baseline apical ATP release under control conditions was defined as 100% (Figure 5). In comparison, ATP detected at the basolateral side was markedly lower. Apical application of the PANX1 inhibitor Brilliant Blue FCF (BB‐FCF) [19] significantly reduced apical ATP release. Pharmacological stabilization of HIF‐1α using ICA markedly enhanced apical ATP secretion, an effect that was abolished by simultaneous apical BB‐FCF treatment. Notably, basolateral application of BB‐FCF did not alter apical ATP release. The PANX1 inhibitor Probenecid significantly decreased apical ATP release regardless of whether it was applied apically or basolaterally, suggesting its cell‐permeable nature enables inhibition across both membrane domains.

### 3.6 Basolateral Application of Probenecid but Not BB‐FCF Inhibits In Vitro Cyst Growth

**FIGURE 6:**
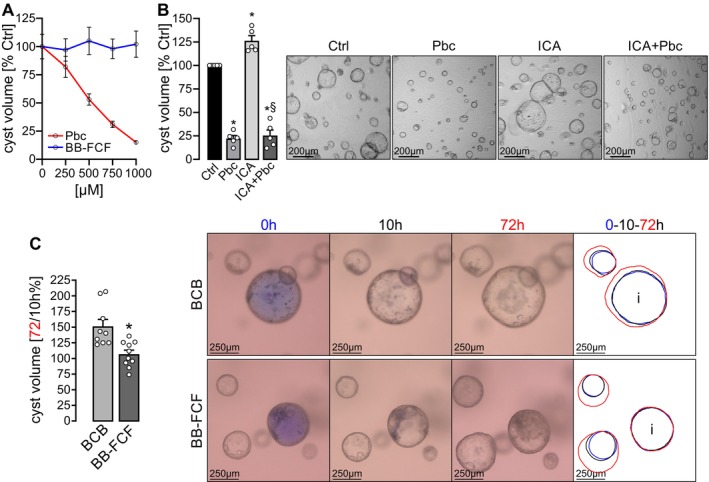
Compartment‐specific inhibition of cyst growth by Probenecid and BB‐FCF. *PKD1* ^−/−^ #1 cells were cultured in a collagen I matrix, forming cysts that significantly expanded over 5 days. (A) Basolateral application of Probenecid (Pbc) via the culture medium reduced cyst growth in a dose‐dependent manner. BB‐FCF, in contrast, had no effect at any tested concentration. (B) Cysts were treated under control conditions (Ctrl) or supplemented with Probenecid (750 μM), ICA (10 μM), or the combination of ICA and Probenecid (ICA + Pbc); each condition *n* = 6. Probenecid significantly inhibited cyst growth and abrogated ICA‐induced cyst enlargement. Representative images show cysts within the collagen matrix at endpoint. *Significant vs. Ctrl; ^§^Significant vs. ICA. (C) After 7 days of culture, cysts were microinjected with either BB‐FCF (100 μM; *n* = 10 from three independent experiments) or brilliant cresyl blue (8.7 μM; *n* = 9 from three independent experiments) as control. Final luminal BB‐FCF concentrations were 4.6 μM (range: 2.1–12.5 μM), and the representative images shown correspond to 3.3 μM BB‐FCF and 0.29 μM BCB in the BB‐FCF‐injected cyst, and to 0 μM BB‐FCF and 0.15 μM BCB in the control‐injected cyst. Live imaging assessed cyst volume increase between 10 and 72 h post‐injection. Images depict representative cysts at injection time (0 h) and at subsequent timepoints (10 h and 72 h). Injected cysts were identified via the visible blue dye. Color‐coded outlines indicate cyst dimensions: Blue (0 h), black (10 h), and red (72 h). Notably, in the BCB panel, both injected (i) and non‐injected cysts showed volume increase, while in the BB‐FCF panel, only non‐injected cysts grew. BB‐FCF‐injected cysts (i) failed to expand further. *Significant vs. BCB.

We next evaluated the effects of the PANX1 inhibitors Probenecid and Brilliant Blue FCF (BB‐FCF) on in vitro cyst growth in *PKD1* ^−/−^ #1 clone cells. Basolateral application of Probenecid significantly inhibited cyst enlargement in a dose‐dependent manner (Figure 6A). In contrast, BB‐FCF had no measurable impact on cyst growth at any tested concentration (Figure 6A). Notably, Probenecid suppressed cyst enlargement not only under basal conditions but also effectively blocked ICA‐induced cyst growth (Figure 6B).

This difference between the two inhibitors is consistent with their distinct physicochemical properties. Probenecid is cell‐permeable and can therefore inhibit PANX1 from both membrane domains. BB‐FCF, in contrast, is cell‐impermeable and inhibits PANX1 exclusively from the extracellular side [23]. Since PANX1 in cyst‐lining epithelial cells is localized to the apical membrane, BB‐FCF requires luminal access to reach its binding site. This rationale motivated the subsequent microinjection experiments, which directly test the effect of apical PANX1 inhibition on cyst growth.

### 3.7 Injection of BB‐FCF Into the Cyst Lumen Inhibits Cyst Enlargement

To test whether luminal application of BB‐FCF inhibits in vitro cyst growth, BB‐FCF was microinjected into the cyst lumen using a pulled glass capillary and precision micromanipulator. Brilliant Cresyl Blue (BCB) served as an injection control. Following BCB injection, cysts exhibited a recovery phase lasting approximately 10 h, after which they resumed growth (Figure S7). Cyst volume was quantified at two time points—10 h (baseline post‐injection) and 72 h—and a clear increase in size was observed in control cysts (Figure 6C). In contrast, injection of BB‐FCF resulted in minimal to no increase in cyst volume between the two time points, indicating a robust suppression of cyst expansion. These findings suggest that luminal delivery of BB‐FCF inhibits PANX1‐mediated ATP release, thereby attenuating cyst growth in *PKD1* ^*−/−*^ cells.

## 4 Discussion

ATP accumulates in the luminal fluid of kidney cysts in autosomal dominant polycystic kidney disease (ADPKD) at concentrations sufficient to activate luminal purinergic receptors, such as P2Y2R [24]. Activation of this Gq‐coupled receptor raises intracellular calcium levels, triggering the calcium‐activated chloride channel TMEM16A (Anoctamin‐1), which promotes cyst expansion [5, 6]. The mechanisms underlying ATP release into the cyst lumen remain incompletely understood and may involve vesicular nucleotide release, as observed in non‐epithelial cells, or passive release from cyst‐lining cells undergoing cell death [25]. Recent research, however, has revealed that kidney tubular cells actively secrete ATP via membrane channels, including connexins, pannexins, LRRC8A, and maxi‐anion channels [21, 26].

In distal tubules and collecting ducts, the primary sites of cystogenesis in ADPKD, apical ATP secretion appears to involve Connexin 30 and PANX1 [27, 28, 29]. PANX1 has garnered particular interest due to its upregulation in *PKD1*‐deficient kidney cells and its role in mediating ATP release under fluid shear stress, as evidenced by inhibition with the PANX1 blocker BB‐FCF [30]. Moreover, administration of Probenecid, which also inhibits PANX1 among other targets, reduces cyst burden in a *Pkd1*‐deficient mouse model [31].

Progressive cyst growth leads to vascular rarefaction and local hypoxia, resulting in the induction of HIF‐1α in the cyst epithelium [8, 32]. HIF‐1α promotes cyst enlargement by stimulating calcium‐activated chloride secretion via TMEM16A [9]. Although TMEM16A is not a direct transcriptional target of HIF‐1α, our findings show that P2Y2R is regulated in a HIF‐dependent manner, linking hypoxic signaling to secretory‐driven cyst growth [6, 7]. We further hypothesized that ATP release into the cyst lumen may also be modulated by HIF‐1α, integrating ATP signaling into the hypoxia‐driven secretory cascade.

Our results identify PANX1 as a direct HIF‐1α target in human primary kidney tubular cells. In addition, PANX1 is abundantly localized to the luminal membrane of cysts in nephrectomy specimens from ADPKD patients. Both in vitro and in vivo, loss of *PKD1* induces PANX1 expression, an effect amplified by pharmacological induction of HIF‐1α. Correspondingly, ATP is released apically in *PKD1*‐deficient tubular cells, and this release is potentiated by HIF‐1α activation and attenuated by the PANX1 inhibitors Probenecid and BB‐FCF. Notably, BB‐FCF, a membrane‐impermeable compound, affects cyst growth only when delivered to the luminal compartment, consistent with its requirement for apical access to PANX1. The clinical relevance of HIF‐1α activation in ADPKD is underscored by the recent introduction of HIF prolyl hydroxylase inhibitors (HIF PHIs) such as roxadustat for the treatment of anemia in chronic kidney disease. We previously demonstrated that pharmacological HIF‐1α stabilization accelerates cyst growth in an orthologous Pkd1 mouse model, raising concerns that systemic HIF activation may exacerbate disease progression in ADPKD [10]. Reflecting this, the current KDIGO guideline for ADPKD advises against the use of HIF PHIs in patients with ADPKD who are not receiving dialysis [33]. Together, these findings suggest that therapeutic HIF activation may have unintended consequences in ADPKD and highlight the need for careful consideration of HIF PHI use in this patient population.

The HIF‐dependent ATP release via PANX1 likely operates alongside other pathways. Connexin 30, also expressed apically in distal nephron segments, may contribute to ATP transport but appears neither HIF‐regulated nor sensitive to PANX1 inhibitors [34]. Additionally, ATP may originate from dying cells; however, necrosis and necroptosis are not characteristic of intact cyst‐lining epithelia and are likely more prominent in inflamed or degenerating cysts. In contrast, regulated forms of cell death, including apoptosis and ferroptosis, are frequently observed [35, 36]. Ferroptotic cells may release ATP passively due to membrane damage, whereas apoptotic cells release ATP in a controlled manner via activated PANX1 channels [37, 38].

Our study has limitations. The demonstration of PANX1 expression in murine and human cyst‐lining epithelia underscores its potential relevance in vivo; however, the data are primarily descriptive. Human tissue samples were almost exclusively derived from end‐stage kidney disease kidneys, limiting generalizability. Functional assays on ATP release and cyst dynamics were conducted in vitro. Moreover, our conclusions rely on pharmacological inhibition of PANX1, as genetic loss‐of‐function approaches were not included in this study. Nonetheless, when integrated with existing literature showing the efficacy of Probenecid in ADPKD mouse models, and our own evidence of HIF‐induced cyst growth and chloride secretion, a coherent model emerges linking HIF‐1α, ATP release, and secretion‐driven cyst enlargement.

In conclusion, our findings demonstrate that HIF‐1α directly induces PANX1‐mediated ATP release from the apical membrane of cyst‐lining epithelial cells in ADPKD. Through this pathway, hypoxia engages purinergic signaling to activate calcium‐dependent chloride secretion, thereby contributing to cyst growth. Targeting PANX1 may offer a promising therapeutic approach to disrupt secretion‐driven cyst expansion.

## Author Contributions

B.B., A.K., and J.S. created the concept and design of the work. A.K., K.S., J.K.S., R.U., S.G., S.N., V.L., and M.B.‐H. performed experiments, A.K., K.S., J.K.S., J.S., S.G., S.N., M.S., and B.B. analyzed data, B.B. wrote the first draft of the manuscript. All authors were involved in drafting and revising the manuscript.

## Funding

This study was supported by Deutsche Forschungsgemeinschaft (DFG) (509149993).

## Disclosure

The authors have nothing to report.

## Conflicts of Interest

The authors declare no conflicts of interest.

## Supporting information


**Figures S1–S7:** fsb271892‐sup‐0001‐FiguresS1‐S7.pdf.

**Table S1:** fsb271892‐sup‐0002‐TablesS1‐S2.docx.
**Table S2:** fsb271892‐sup‐0002‐TablesS1‐S2.docx.

**Appendix S1:** fsb271892‐sup‐0003‐AppendixS1.pdf.

## Data Availability

This paper does not report original code. Any additional information required to reanalyze the data reported in this paper is available from the corresponding author, upon request.
