# Supplementary material for: HIF‐Regulated Pannexin‐1 Channel Drives Luminal ATP Accumulation in Kidney Cysts
Source: FASEB J. 2026 May 12;40:e71892. doi: 10.1096/fj.202502847RR (PMC13164829; doi:10.1096/fj.202502847RR)

HIF-regulated Pannexin-1 Channel Drives Luminal ATP Accumulation in Kidney Cysts

Supplement

Supplemental Figure 1. HIF does not interact with intragenic regions of *LRRC8A, GJB6,* or *TMEM16A.* Shown are the genomic loci of **A** *LRRC8A*, **B** *GJB6*, **C** *EGLN3* (a well-characterized HIF-1 target gene), and **D** *TMEM16A*. Conserved binding of both HIF subunits is highlighted in red and is observed only within intragenic regions of *EGLN3*. For *TMEM16A*, no HIF-1α or HIF-1β binding was detected within the genomic region covered by our dataset. While this does not exclude the possibility of more distal regulatory elements, the functional HIF-dependent activation of calcium-activated chloride secretion is more plausibly explained by upstream mechanisms, including HIF-dependent regulation of *PANX1*.

Supplemental Figure 2. PANX1 expression in human ADPKD kidneys (recombinant monoclonal antibody). Kidney specimens from 29 ADPKD patients (male: n = 19; female: n = 10) undergoing nephrectomy were analyzed. Representative tissue microarray (TMA) samples from patients 1-15 are shown, stained with a recombinant rabbit monoclonal anti-PANX1 antibody (Thermo Fisher Scientific, MA5-50140). PANX1 immunoreactivity appears in cyst-lining epithelial cells (brown), counterstained with hematoxylin.

Supplemental Figure 3. PANX1 expression in human ADPKD kidneys (recombinant monoclonal antibody). Representative TMA samples from patients 16-29 stained with the recombinant rabbit monoclonal anti-PANX1 antibody (Thermo Fisher Scientific, MA5-50140). PANX1 staining (brown) highlights cyst-lining epithelial cells; hematoxylin counterstain.

Supplemental Figure 4. PANX1 expression in human ADPKD nephrectomies (polyclonal antibody). Kidney specimens from 29 ADPKD patients (male: n = 19; female: n = 10) were analyzed using a polyclonal rabbit anti-PANX1 antibody (Abcam, ab139715). Clinical characteristics are summarized in Table 1. **A** Quantification of PANX1 staining expressed as positive area per tissue area; each data point represents the mean of three samples per patient. **B** Representative TMA sample stained for PANX1 (brown). Magnified regions (1-2) highlight luminal/apical staining in cyst-lining epithelial cells (arrows), partially obscured by additional nuclear staining characteristic of this antibody.

Supplemental Figure 5. PANX1 expression in human ADPKD kidneys (polyclonal antibody). Representative TMA samples from patients 1-15 stained with the polyclonal rabbit

anti-PANX1 antibody (Abcam, ab139715). PANX1 staining (brown) and hematoxylin counterstain are shown.

Supplemental Figure 6. PANX1 expression in human ADPKD kidneys (polyclonal antibody). Representative TMA samples from patients 16-29 stained with the polyclonal rabbit anti-PANX1 antibody (Abcam, ab139715). PANX1 staining (brown) and hematoxylin counterstain are shown.

Supplemental Figure 7. Early post-injection stabilization phase defines 10 h as the earliest biologically meaningful reference time point (T1). Cysts derived from *PKD1*$^{-/-}$ #1 cells were cultured in collagen I and microinjected with either brilliant cresyl blue (BCB, control) or BB-FCF (PANX1 inhibitor), as described in Figure 6C. Time-lapse imaging was performed from 0 h until 72 h post-injection. Across all conditions, cysts exhibited a characteristic stabilization phase during the first 10 hours after puncture, dominated by mechanical effects of the injection procedure.

BCB 01: Cyst size remained unchanged between 0 h and 10 h, as indicated by the grey reference band marking the upper and lower poles of the cyst. After 10 h, the cyst grows continuously until 72 h.

BCB 02: A transient decrease in cyst size occurred immediately after injection, followed by stabilization by approximately 10 h and subsequent growth up to 72 h.

BB-FCF 01: Cyst size remained stable during the first 10 h, similar to BCB controls, but did not increase thereafter.

BB-FCF 02: A modest early decrease in cyst size was observed up to 10 h, followed by stable cyst dimensions throughout the 72 h observation period.

Since the interval from 0-10 h reflects injection-related mechanical effects rather than biological growth or inhibitor action, 10 h represents the earliest reproducible and biologically meaningful reference time point (T1) for quantifying subsequent cyst expansion.

Supplemental Figure 1

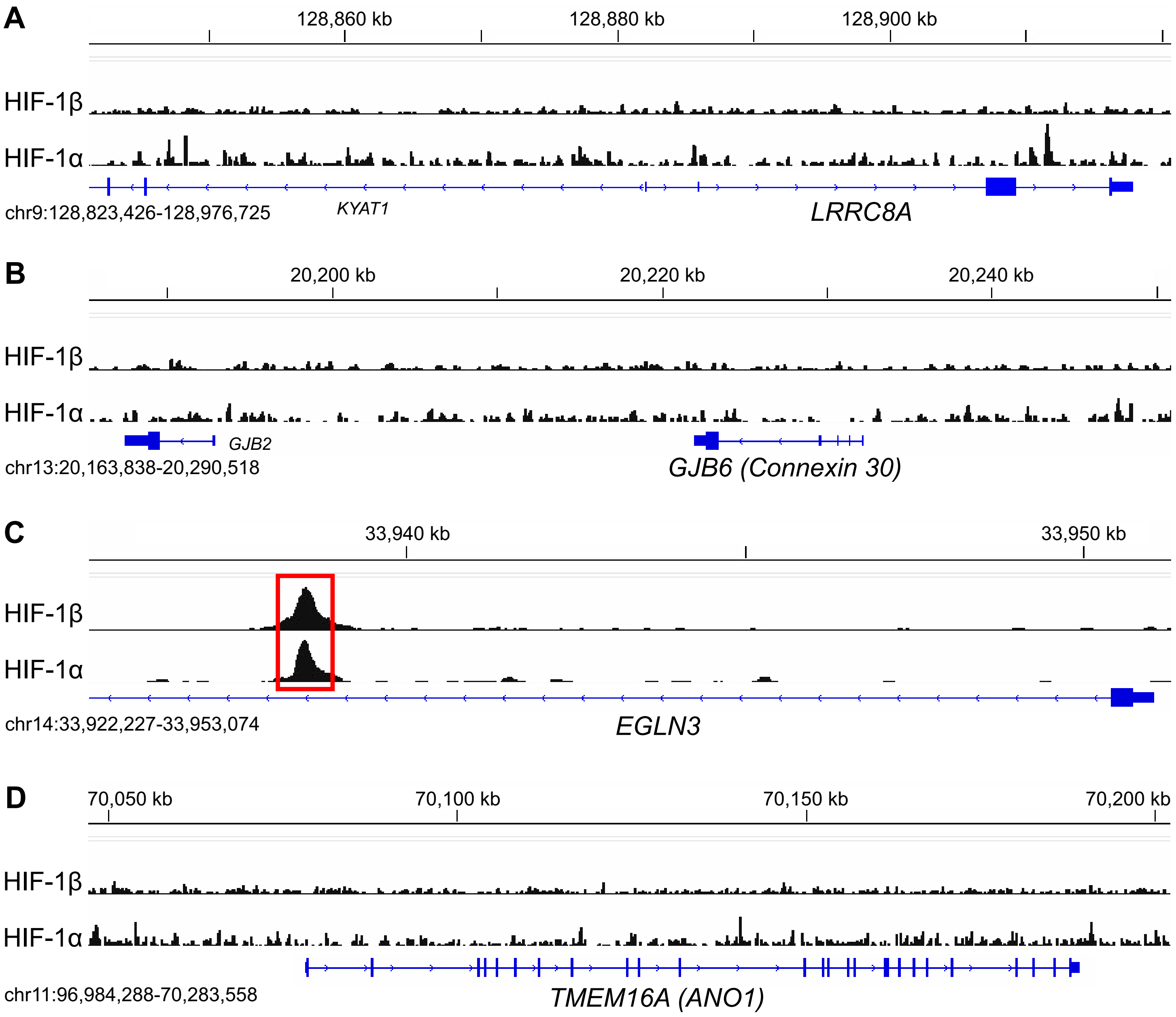

Supplemental Figure 2

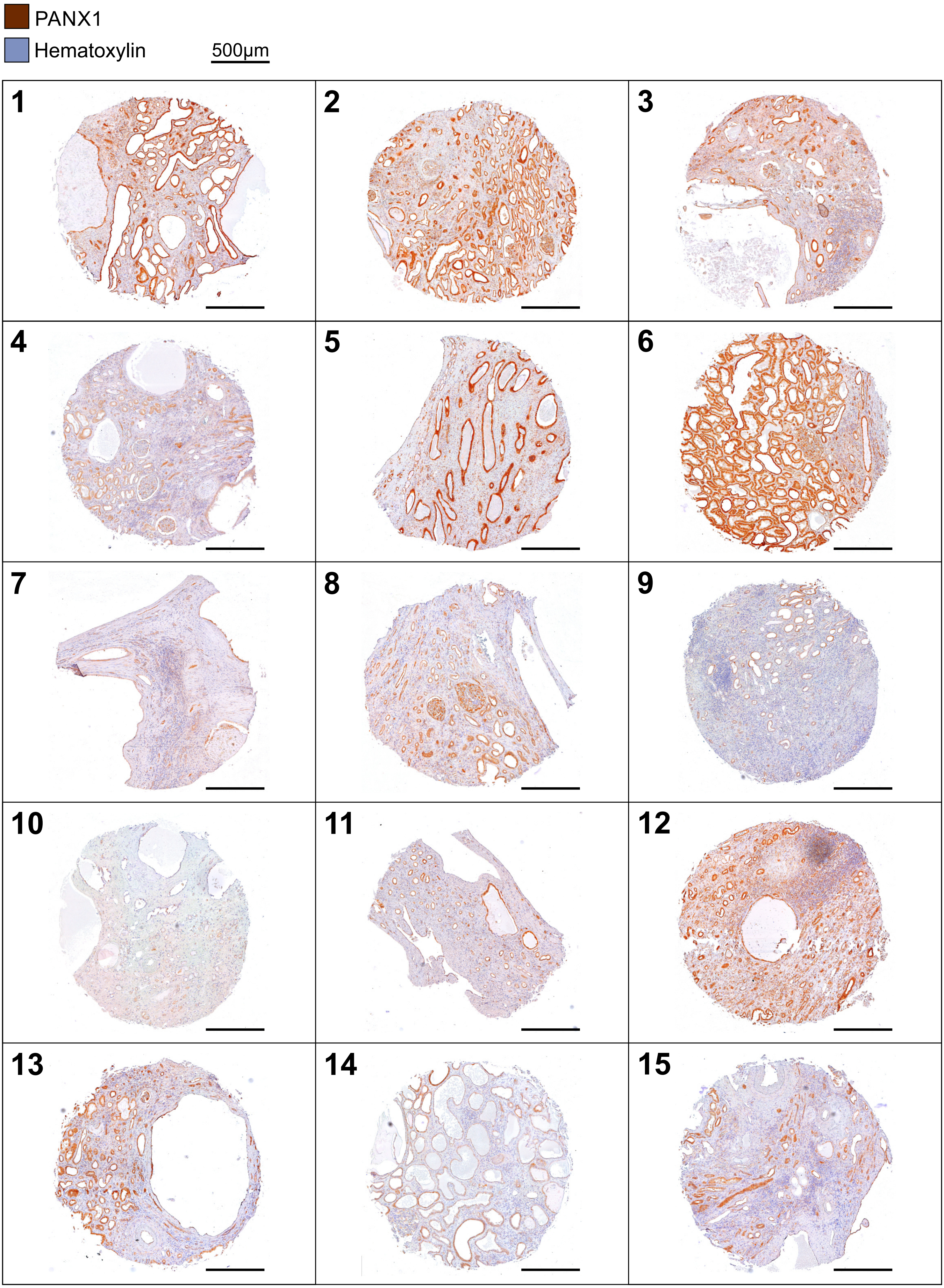

Supplemental Figure 3

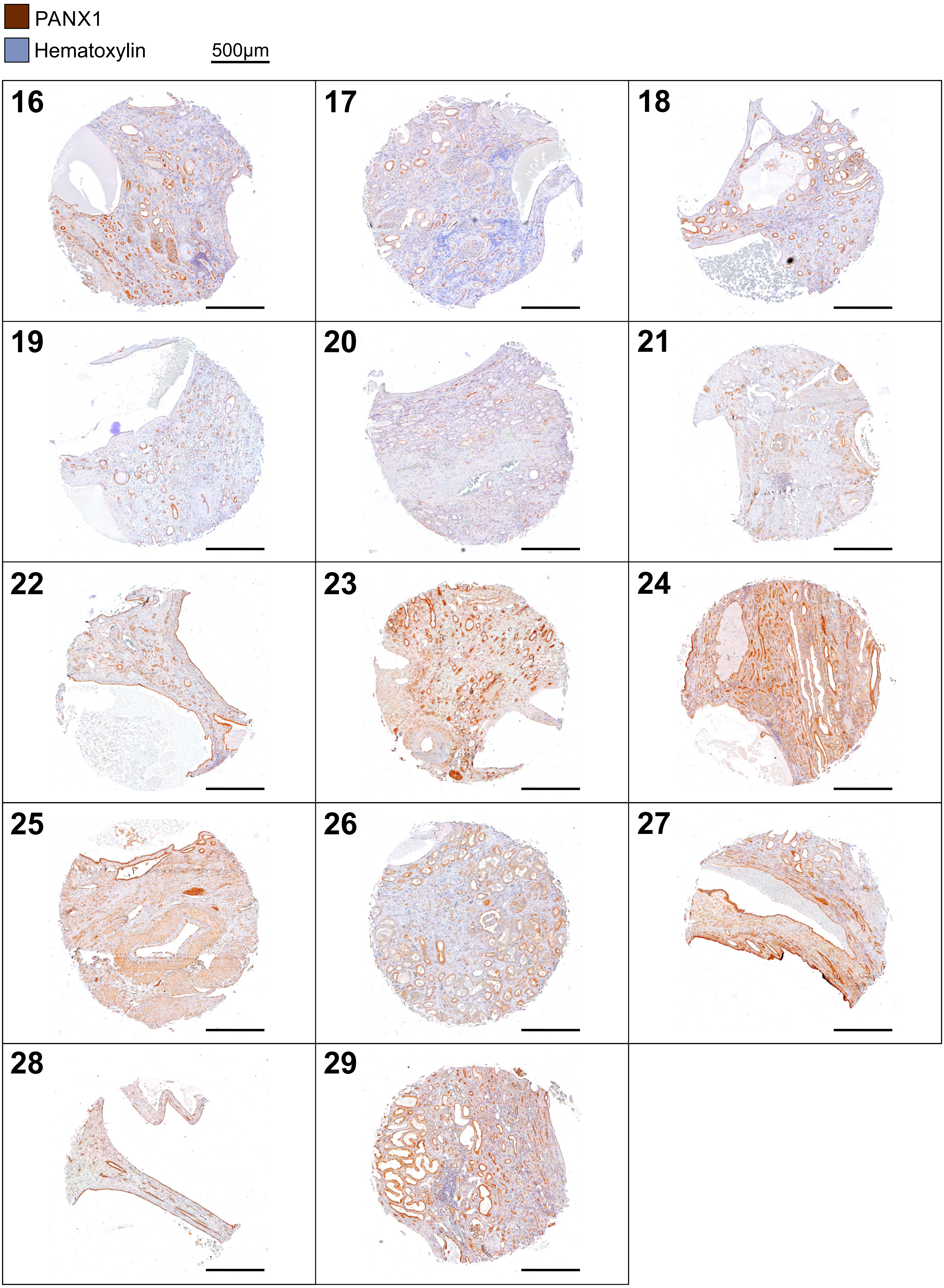

Supplemental Figure 4

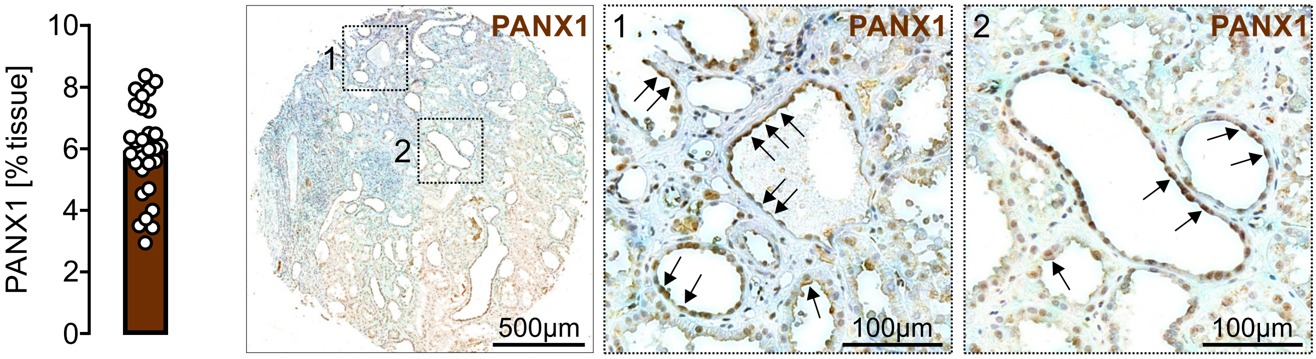

Supplemental Figure 5

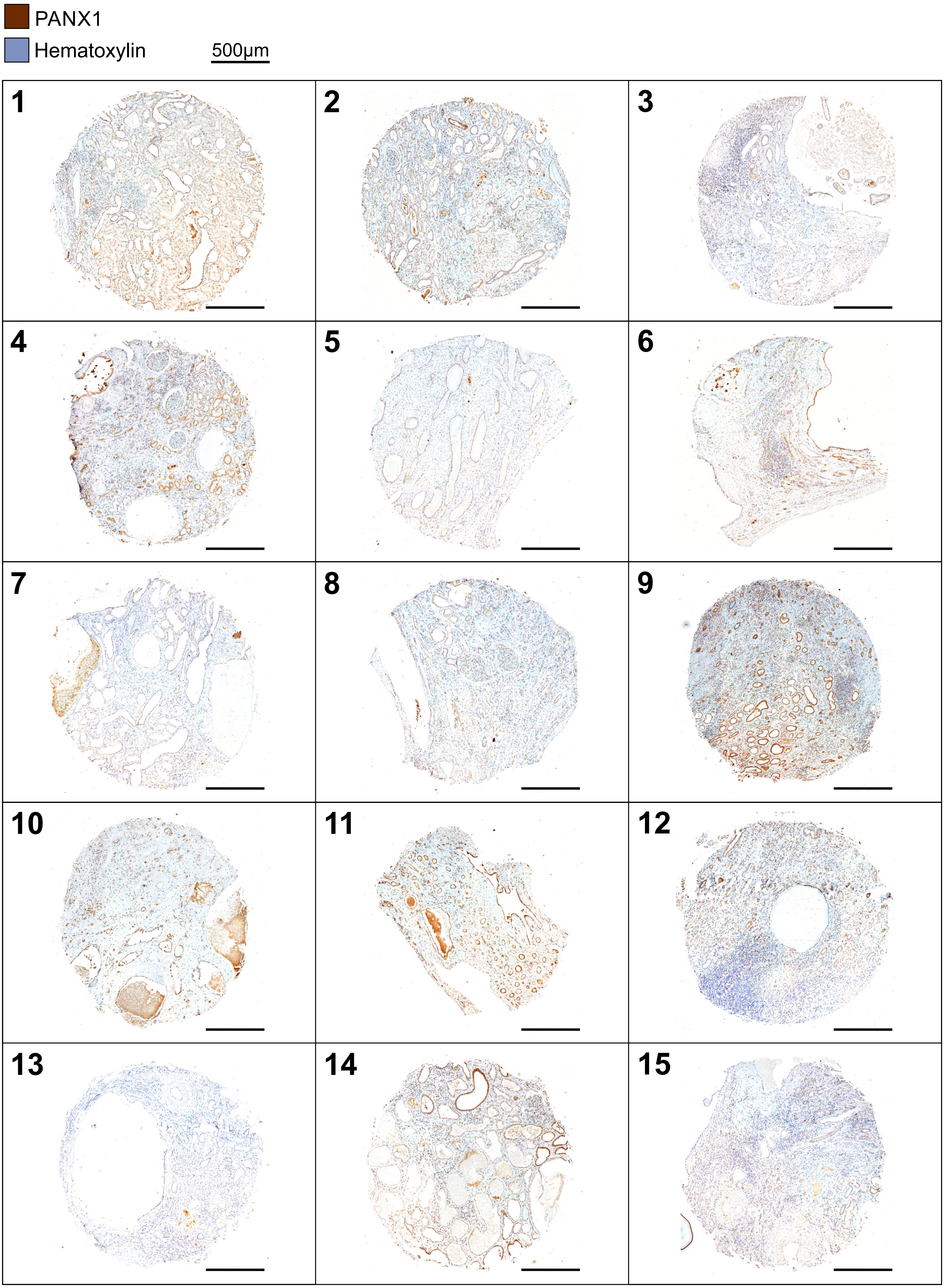

Supplemental Figure 6

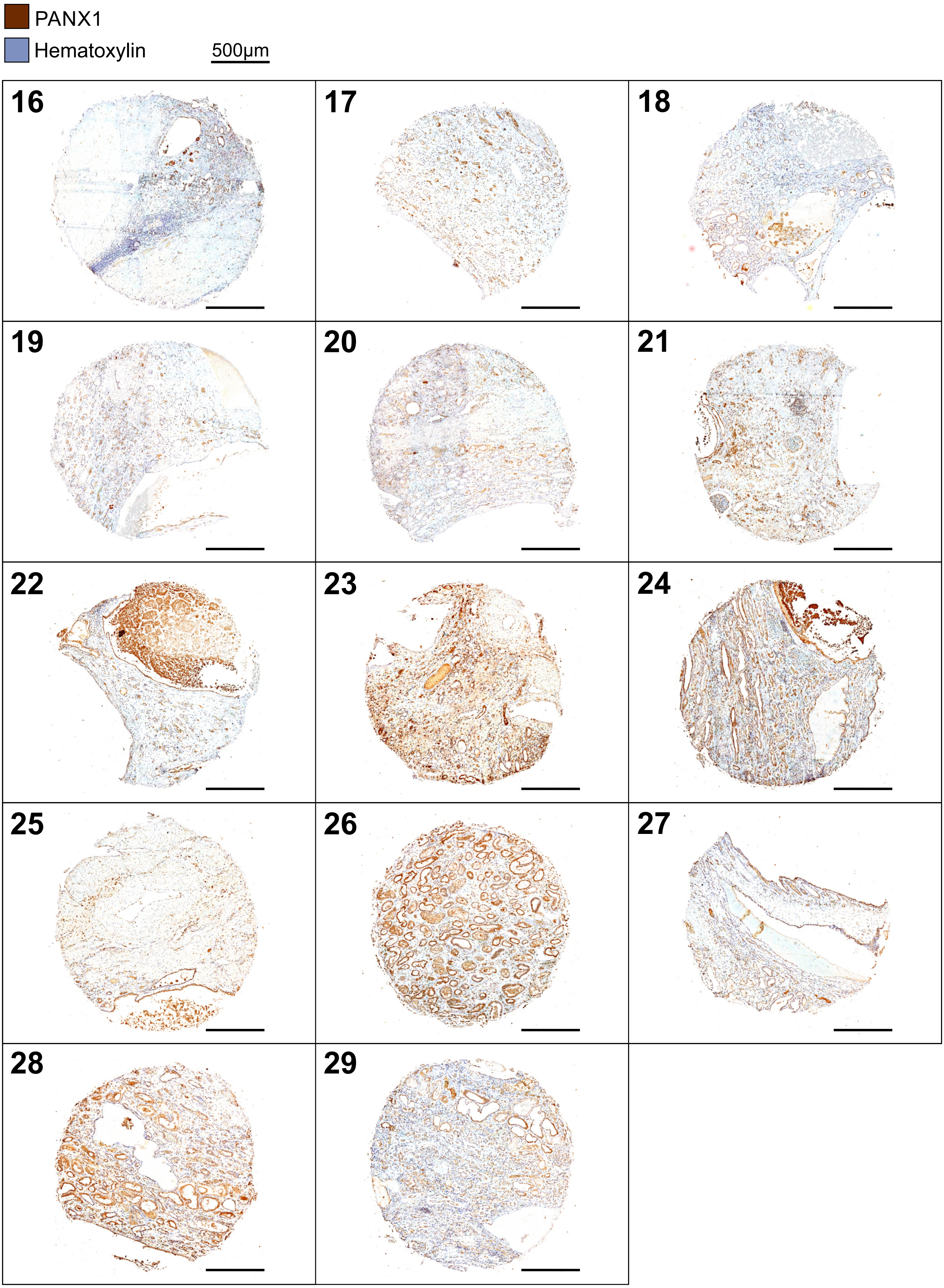

Supplemental Figure 7

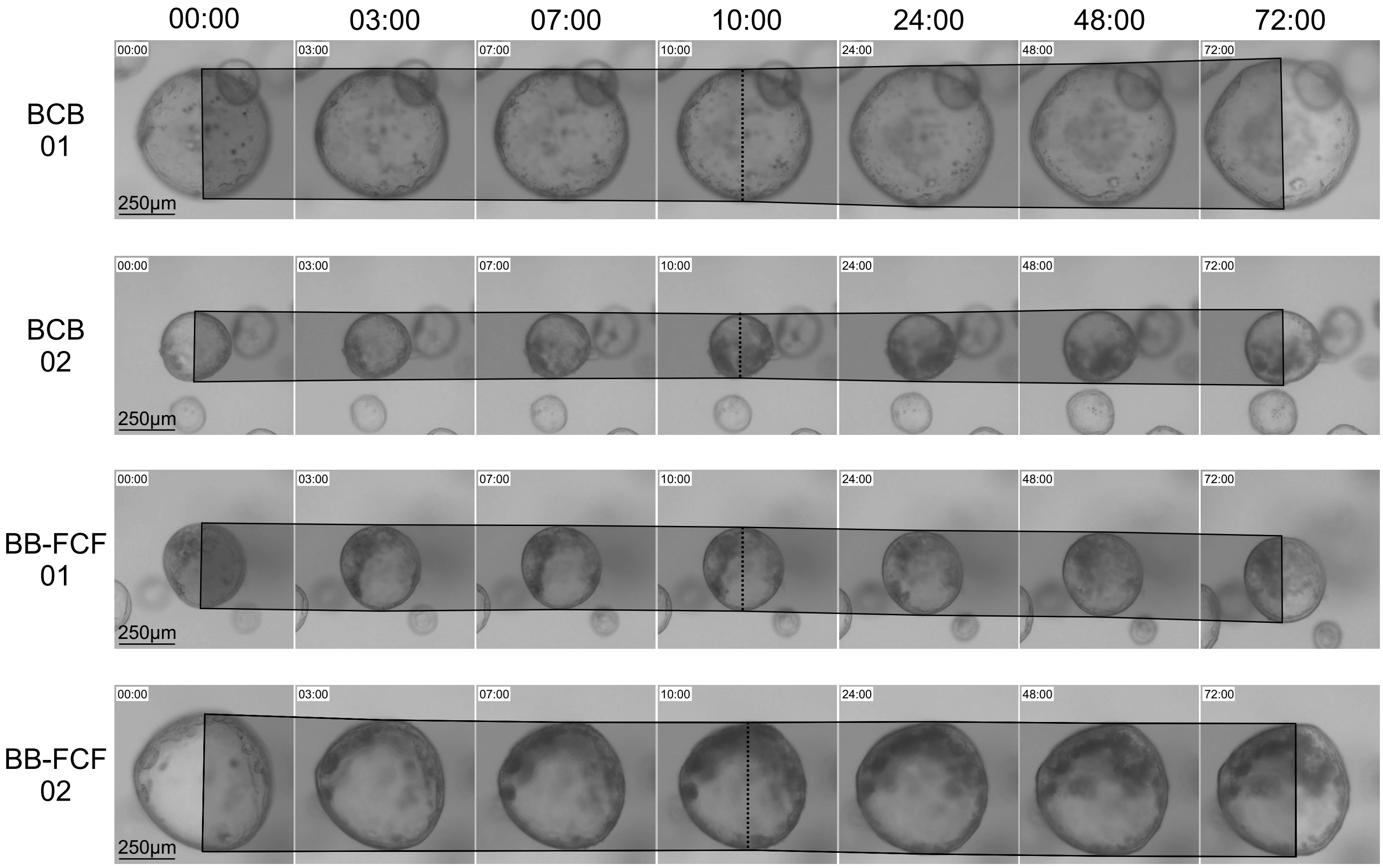

Supplement: Supplementary file 1 — Figures S1–S7: fsb271892‐sup‐0001‐FiguresS1‐S7.pdf. [file FSB2-40-e71892-s003.pdf]
