# Supplementary material for: HIF‐Regulated Pannexin‐1 Channel Drives Luminal ATP Accumulation in Kidney Cysts
Source: FASEB J. 2026 May 12;40:e71892. doi: 10.1096/fj.202502847RR (PMC13164829; doi:10.1096/fj.202502847RR)
Supplement: Supplementary file 2 — Table S1: fsb271892‐sup‐0002‐TablesS1‐S2.docx. Table S2: fsb271892‐sup‐0002‐TablesS1‐S2.docx. [file FSB2-40-e71892-s001.docx]

**HIF-regulated Pannexin-1 Channel Drives Luminal ATP Accumulation in Kidney Cysts**

**Supplemental Table 1: Real-time Primer sequences**

| **species** | **gene** | **sequence** |
| --- | --- | --- |
| Human | *HPRT* | GACCAGTCAACAGGGGACAT (fw)  AACACTTCGTGGGGTCCTTTTC (rev) |
| Human | *EGLN3* | GGCCATCAGCTTCCTCCTG (fw)  GGTGATGCAGCGACCATCA (rev) |
| Human | *PANX1* | AAGATGGTCACGTGCATTGC (fw)  TGACGCCAGGAGAAAGAACT (rev) |

**Supplemental Table 2. siRNA sequences**

| dHIF | CCUACAUCCCGAUCGAUGAtt (sense)  UCAUCGAUCGGGAUGUAGGtt (antisense) |
| --- | --- |
| HIF-1α | CUGAUGACCAGCAACUUGAtt (sense)  UCAAGUUGCUGGUCAUCAGt (antisense) |
| HIF-1β | ON-TARGETplus Human ARNT (405) siRNA –  SMARTpool (L-007207-00-0005) |
